# Functional polymorphisms in the promoter regions of MMP2 and MMP3 are not associated with melanoma progression

**DOI:** 10.1186/1477-5751-6-9

**Published:** 2007-10-24

**Authors:** Javier Cotignola, Pampa Roy, Ami Patel, Nicole Ishill, Shivang Shah, Alan Houghton, Daniel Coit, Allan Halpern, Klaus Busam, Marianne Berwick, Irene Orlow

**Affiliations:** 1 Epidemiology and Biostatistics, Memorial Sloan-Kettering Cancer Center, New York, NY, USA; 2 Department of Medicine, Memorial Sloan-Kettering Cancer Center, New York, NY, USA; 3 Department of Surgery, Memorial Sloan-Kettering Cancer Center, New York, NY, USA; 4 Pathology Department, Memorial Sloan-Kettering Cancer Center, New York, NY, USA; 5 Division of Epidemiology, University of New Mexico, Albuquerque, NM, USA

## Abstract

**Background:**

The matrix metalloproteinases (MMPs) are enzymes that cleave various components of the extracellular matrix (ECM) and basement membranes. MMPs are expressed in melanocytes and their overexpression has been linked to tumor development, progression and metastasis. At the genetic level, the following functional promoter polymorphisms are known to modify the gene transcription: -1306 C/T and -735 C/T in the MMP2 gene, and -1171 5A/6A in the MMP3 gene. Functional polymorphisms in MMP genes' promoter regions may modulate the risk for melanoma progression.

**Methods:**

We evaluated MMP2 and MMP3 germline polymorphisms in a group of 1002 melanoma patients using PCR-based methods, including fragment size analysis and melting temperature profiles. Two-sided Chi-Square, Cochran-Armitage tests for trend, Fisher's exact tests, and Kendall's Tau tests were performed to evaluate the associations between genotype and various clinical and epidemiologic factors. Multivariate analyses were conducted using logistic regression, adjusting for known melanoma confounders such as age, sex, phenotypic index, moles, freckles, and race. Survival estimates were computed using the Kaplan-Meier method and differences in survival were assessed using the log rank test.

**Results:**

All genotypes were in Hardy-Weinberg equilibrium. After adjustment for age, sex and phenotypic characteristics of melanoma risk, no significant associations were identified with the clinical, pathological, and epidemiological variables studied. The melting profile for MMP2 -735 C/T identified a new change in one sample. A new PCR-amplification followed by direct sequencing confirmed a heterozygote G to A substitution at position -729.

**Conclusion:**

This study does not provide strong evidence for further investigation into the role of the MMP2 and MMP3 variants in melanoma progression.

## Background

The matrix metalloproteinases (MMPs) are enzymes that cleave various components of the extracellular matrix (ECM) and basement membranes. Upon degradation, the ECM releases and activates ECM-bound cytokines and ECM fragments which modulate cell growth and migration as well as angiogenesis [1].

MMPs are expressed in melanocytes and their overexpression has been linked to tumor development, progression and metastasis [2-5]. Certain MMPs are associated with generalized growth and expansion of the cell mass while others are involved in *in situ *tumor progression, invasion of microvasculature, and metastasis [6]. Nikkola *et al*. tested the expression of MMPs in 56 metastatic melanomas by immunohistochemistry and found that patients with positive tumors for MMP1 and MMP3 had a shorter disease-free survival when compared to those with negative lesions (MMP1, p = 0.0383; MMP3, p = 0.0294) [7]. In another study, investigators have found strong expression (> 40% cells stained) of MMP2 in 78% of the invasive melanomas [8].

At the genetic level, two functional promoter single nucleotide polymorphisms (SNPs) have been described in the MMP2 gene [rs243865: -1306 C/T; and rs2285053: -735 C/T], and one functional insertion/deletion in the promoter region of the MMP3 gene [rs3025058: -1171 5A/6A]. All changes produce either a disruption or creation of binding sites for transcriptional regulators which modify the gene transcription and, in turn, the enzymatic levels [9-11]. Specifically, for MMP2 both C to T transitions disrupt Sp1 binding sites and, consequently, decrease the transcription rate [9,11]. For MMP3, the insertion of an A at position -1171 allows for the binding of a transcriptional repressor [10].

Functional SNPs in MMP genes' promoter regions may modify the production of proteolytic enzymes, and in turn modify the risk for melanoma progression. Therefore, in this study, we sought to determine whether an association between MMP2 and MMP3 SNPs and disease progression exists. Functional promoter polymorphisms in MMP2 and MMP3 genes were examined in a cohort of 1002 melanoma patients.

## Results

This study included 1002 melanoma patients with stages 0 (*in situ*) to IV. Nine hundred and forty eight (95%) were cutaneous malignant melanoma (CMM) patients; the rest included mucosal melanomas (n = 11), other non-cutaneous sites (n = 1) and unknown primary sites (n = 42). Ninety-six percent were Caucasians followed by Hispanic (1.1%), black non-Hispanic (1.1%), and Asian/Indian (0.3%); fifteen patients had missing information on ethnicity and one declined to answer the question about race (1.5%). The age at diagnosis ranged from 5 to 89 years old (mean = 54 and median = 55). The genotyping success rate was in the range from 98.2 to 99.5% and the retesting of the 10% randomly selected samples was 100% concordant.

### MMP2 -1306 C/T and -735 C/T

**Figure 1:**
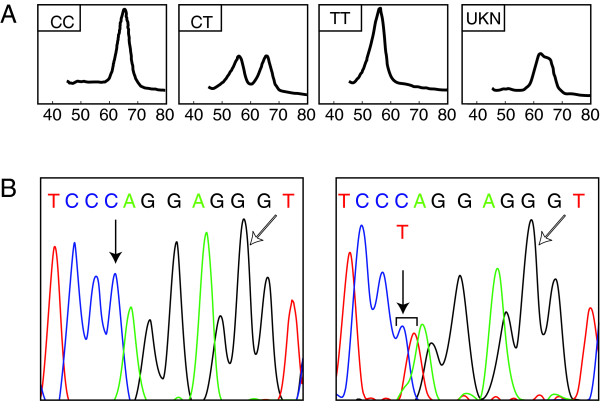
**Genotyping of MMP2 -735 C/T SNP by melting temperature analysis**. **(A) **The top panels depict the derivative melting curve plots obtained for (from left to right): MMP2 -735CC, MMP2 -735CT, MMP2 -735TT, and an unexpected profile (UKN) showing peaks between 62 and 66°C **(B) **The bottom panel depicts the wild type (left) and a new variant (right) at position -729 near the target SNP (white arrow). Reverse sequencing revealed a novel mutation corresponding to a G to A change at position -729 in the sense strand (black arrow).

One sample showed an unexpected profile in the melting temperature analysis of -735 C/T that did not match any of the three possible genotypes (Figure 1). The direct sequencing on this sample showed a heterozygote G to A substitution at position -729 [ss_49785040], and the homozygote wild type C allele at position -735. The analysis with the UCSC Genome Browser did not show any conserved sequence within the region bearing the new variant [12].

The allele frequencies for MMP2 -1306 C/T were similar to those reported in the dbSNP for the Caucasian population CAUC1 [13]. The frequencies for MMP2 -735 C/T were also similar to those describe in the dbSNP, even though these data were only based on a Japanese population. Both genotypes were in Hardy-Weinberg equilibrium. When compared to the patients' number of moles, the -1306TT genotype was more frequent among those patients with 'many moles' (p < 0.01); however, after adjustment for age, sex, phenotypic index, freckles, and race this association was no longer significant (Table 1). No other significant associations were found between the MMP2 SNPs and the phenotypic and clinico-pathological melanoma features.

**Table 1:** MMP2 genotypes *vs *clinical, pathological and epidemiological variables

**Variables**	**MMP2**							
	
	**-1306 C/T**			p-value*	**-735 C/T**			p-value*
				
	**CC**	**CT**	**TT**		**CC**	**CT**	**TT**	
***Stage at Diagnosis***								
0	34 (59%)	19 (33%)	5 (9%)		48 (83%)	10 (17%)	0 (0%)	
I	320 (64%)	159 (32%)	23 (5%)		365 (73%)	123 (25%)	13 (3%)	
II	152 (65%)	72 (31%)	9 (4%)		184 (78%)	49 (21%)	2 (1%)	
III	105 (63%)	54 (32%)	9 (5%)		131 (79%)	32 (19%)	3 (2%)	
IV	6 (67%)	3 (33%)	0 (0%)	0.92	4 (44%)	5 (56%)	0 (0%)	0.08
***Primary Clark Level***								
I	34 (59%)	19 (33%)	5 (9%)		48 (83%)	10 (17%)	0 (0%)	
II	70 (65%)	36 (33%)	2 (2%)		79 (74%)	23 (22%)	5 (5%)	
III	89 (60%)	48 (32%)	12 (8%)		115 (77%)	32 (21%)	3 (2%)	
IV	308 (64%)	155 (32%)	22 (5%)		358 (74%)	116 (24%)	10 (2%)	
V	51 (74%)	14 (20%)	4 (6%)	0.20	54 (78%)	15 (22%)	0 (0%)	0.44
***Thickness (mm)***								
*In situ*	34 (59%)	19 (33%)	5 (9%)		48 (83%)	10 (17%)	0 (0%)	
< 1.01	201 (63%)	107 (33%)	12 (4%)		237 (74%)	72 (23%)	10 (3%)	
1.01 – 2.00	174 (64%)	81 (30%)	15 (6%)		199 (73%)	68 (25%)	4 (2%)	
2.01 – 4.00	100 (63%)	50 (31%)	10 (6%)		124 (78%)	33 (21%)	2 (1%)	
> 4.00	81 (68%)	35 (29%)	4 (3%)	0.72	94 (78%)	25 (21%)	1 (1%)	0.44
***TILs***								
Absent	129 (63%)	67 (33%)	9 (4%)		161 (79%)	40 (20%)	3 (2%)	
Non-brisk	262 (67%)	111 (28%)	21 (5%)		289 (73%)	95 (24%)	10 (3%)	
Brisk	19 (63%)	9 (30%)	2 (7%)	0.80	21 (68%)	10 (32%)	0 (0%)	0.32
***Distant Metastasis***								
Yes	86 (67%)	39 (30%)	4 (3%)		97 (76%)	29 (23%)	2 (2%)	
No	543 (63%)	281 (32%)	42 (5%)	0.28	653 (75%)	196 (23%)	17 (2%)	0.88
***Intransit Metastasis***								
No	590 (63%)	302 (32%)	46 (5%)		705 (75%)	216 (23%)	16 (2%)	
Yes	18 (67%)	9 (33%)	0 (0%)	0.44	22 (82%)	3 (11%)	2 (7%)	0.96
***Number of Moles***								
None	161 (62%)	84 (33%)	13 (5%)		201 (77%)	54 (21%)	5 (2%)	
Few	311 (63%)	166 (33%)	20 (4%)		380 (77%)	105 (21%)	11 (2%)	
Moderate	112 (67%)	51 (30%)	5 (3%)		115 (69%)	49 (30%)	2 (1%)	
Many	22 (55%)	11 (28%)	7 (18%)	**< 0.01**	29 (73%)	10 (25%)	1 (3%)	0.40
***Phenotypic Index***								
1 (low risk)	26 (68%)	11 (29%)	1 (3%)		31 (82%)	6 (16%)	1 (3%)	
2	132 (63%)	66 (32%)	11 (5%)		156 (75%)	47 (23%)	4 (2%)	
3	206 (65%)	93 (29%)	18 (6%)		231 (73%)	76 (24%)	10 (3%)	
4	191 (59%)	122 (38%)	11 (3%)		254 (78%)	68 (21%)	4 (1%)	
5 (high risk)	73 (70%)	27 (26%)	5 (5%)	0.28	76 (73%)	28 (27%)	0 (0%)	0.36

* The associations were examined in three different ways (see statistical methods for a detailed explanation). The p-values shown refer to the analysis of the three individual genotypes, and appear in bold font if ≤ 0.05. The significance was lost after adjustment for age, sex, phenotypic index, moles, freckles, and race.

### MMP3 -1171 5A/6A

The MMP3 genotypes were in Hardy-Weinberg equilibrium. In our population, this polymorphism showed a similar distribution to the one reported in the dbSNP for the Italian panel, but the inverse distribution was seen when compared to the PGA-European panel. Although this difference was not significant (p = 0.73). The 5A allele was more common in patients with low Clark level melanomas (p = 0.04) and with tumors with infiltrating lymphocytes (p = 0.04) (Table 2). No associations were seen when we adjusted for confounders.

**Table 2:** MMP3 genotypes *vs *clinical, pathological and epidemiological variables

**Variables**	**MMP3 -1171 5A/6A**			
	**5A5A**	**5A6A**	**6A6A**	p-value*
***Stage at Diagnosis***				
0	9 (16%)	36 (63%)	12 (21%)	
I	96 (19%)	238 (48%)	162 (33%)	
II	32 (14%)	124 (54%)	75 (33%)	
III	27 (16%)	81 (49%)	57 (35%)	
IV	2 (22%)	5 (56%)	2 (22%)	0.36
***Primary Clark Level***				
I	9 (16%)	36 (63%)	12 (21%)	
II	22 (21%)	54 (51%)	29 (28%)	
III	28 (19%)	73 (49%)	47 (32%)	
IV	72 (15%)	241 (50%)	165 (35%)	0.36
V	9 (13%)	39 (57%)	21 (30%)	**0.04^£^**
***Thickness (mm)***				
*In situ*	9 (16%)	36 (63%)	12 (21%)	
< 1.01	60 (19%)	150 (48%)	105 (33%)	
1.01 – 2.00	45 (17%)	132 (49%)	90 (34%)	
2.01 – 4.00	28 (18%)	78 (49%)	53 (33%)	
> 4.00	12 (10%)	71 (60%)	35 (30%)	0.16
***TILs***				
Absent	28 (14%)	101 (50%)	73 (36%)	
Non-brisk	65 (17%)	208 (54%)	114 (30%)	0.32
Brisk	4 (13%)	20 (65%)	7 (23%)	**0.04^£^**
***Distant Metastasis***				
Yes	21 (16%)	69 (54%)	39 (30%)	
No	148 (17%)	428 (50%)	277 (33%)	0.84
***Intransit Metastasis***				
No	160 (17%)	468 (51%)	296 (32%)	
Yes	6 (22%)	12 (44%)	9 (33%)	0.80
***Number of Moles***				
None	41 (16%)	136 (53%)	79 (31%)	
Few	87 (18%)	240 (49%)	165 (34%)	
Moderate	27 (17%)	87 (54%)	48 (30%)	
Many	9 (23%)	21 (53%)	10 (25%)	0.76
***Phenotypic Index***				
1 (low risk)	6 (16%)	15 (41%)	16 (43%)	
2	28 (14%)	105 (51%)	73 (35%)	
3	59 (19%)	157 (50%)	98 (31%)	
4	59 (18%)	172 (54%)	89 (28%)	
5 (high risk)	18 (18%)	47 (46%)	38 (37%)	0.36

*The associations were examined in three different ways (see statistical methods for a detailed explanation). The p-values shown refer to the analysis of the three individual genotypes, and appear in bold font if ≤ 0.05. The significance was lost after adjustment for age, sex, phenotypic index, moles, freckles, and race.

^£^Genotypes 5A6A and 6A6A combined.

We did not find any significant cumulative effect between the number of high-activity alleles and the clinical and epidemiological variables, except for ulceration. Presence of ulceration occurred less frequently in individuals with higher numbers of alleles (p = 0.03). None of the polymorphisms showed associations with progression, survival and recurrence when we computed the Kaplan-Meier estimates and compared differences in survival based on genotypes using a log rank test (data not shown).

## Discussion

After adjustment for age, sex, race, phenotypic index, and freckles, we did not observe any significant associations between the matrix metalloproteinase 2 or matrix metalloproteinase 3 polymorphisms and the clinicopathological and epidemiological variables studied.

Associations between polymorphisms in MMPs and risk of development or progression of the disease have been previously reported in various types of malignancies, including esophageal, breast, and lung cancers [11,14,15] although in some cases the association was not evident perhaps due to the ethnicity and number of cases studied [16]. For MMP2, a case-control study showed that patients with esophageal squamous cell carcinoma carrying the -1306CC or -735CC genotypes had an increased risk of developing cancer (odds ratio (OR) = 1.52, 95% confidence interval (CI) = 1.17–1.96; and OR = 1.30, 95% CI = 1.04–1.63 respectively). A stronger association was seen when individuals with the C_-1306_-C_-735 _haplotype were compared to subjects with the T_-1306_-T_-735 _haplotype (OR = 6.53; 95% CI = 2.78–15.33) [11]. The MMP3 5A allele was associated with a poorer prognosis in breast cancer patients [14]. Su *et al *found no associations between individual MMP1, 3, and 12 and risk of lung cancer although haplotyping revealed a higher risk among never smokers (adjusted OR 3.65, 95% CI:1.62–8.20)[15].

Our genotyping analysis identified a new G to A variation in the MMP2 promoter at position -729. This new variation is not situated within a conserved sequence; therefore, it might not have a functional consequence on the regulation of transcription of MMP2. Even though the functional potential of this nucleotide substitution remains to be determined by *in vitro *assays, the low frequency (0.1%) found in the present study, does not preclude further characterization.

The MMP3 allele with high transcriptional activity 5A was found more frequently in patients with melanomas containing infiltrating lymphocytes and showing low Clark level lesions. It is of note that we are unable to verify whether the missingness of data on TILs in this study is informative therefore these results should be considered preliminary. Although it seems to be a trend between this SNP and TILs and Clark level, the associations lose significance after adjusting for age, sex, phenotypic index, moles, freckles, and race.

## Conclusion

The MMP2 and MMP3 transcriptionally more active polymorphisms appear more frequently among individuals with many moles, tumor infiltrating lymphocytes, and low Clark level, and the number of alleles seems associated with absence of ulceration. However, after controlling for known melanoma confounders the associations are non-significant. This study does not provide strong evidence for further investigation of MMP2 and MMP3 genetic variants in melanoma progression.

## Methods

### Study population

Melanoma patients with stages 0 to IV were recruited at Memorial Hospital (New York, USA) between March 1974 and August 2005. Of these, 763 were newly diagnosed at Memorial Hospital and 239 were prevalent cases. The study protocol was approved by the Memorial Sloan-Kettering Cancer Center (MSKCC) Institutional Review Board (IRB). Ninety-six percent of the patients approached agreed to participate in the study and signed an informed consent. Patients filled out a short self-administered questionnaire that included information on gender, race, age, family history, moles and freckling pattern, nevus density, hair and eye color, propensity to sunburn and ability to tan after sun exposure. The information on hair color, eye color and propensity to tan or sunburn were combined into a single variable, the 'phenotypic index' [17]. This index, with minimum and maximum values of 1 and 5, represents the sum of points assigned to the following phenotypic features: hair color (1 if brown/black; 2 if light brown/blond; 3 if red/auburn); eye color (0 if brown; 1 if green/hazel/blue); and propensity to tan or sunburn (0 if tend to tan; 1 if tend to sunburn). We also obtained clinicopathological information including presence of dysplastic nevi, multiple primary tumors, stage at diagnosis and at follow-up (based on the AJCC 2002 classification), disease status, disease progression and survival among others. The median follow-up period was 40 months (range, 1–493 months, n = 1000). The characteristics of the study group are shown in Table 3.

**Table 3:** Clinico-pathological characteristics of the study group

Variable*	**Patients**	**%**
***Gender***		
Males	573	57.2
Females	429	42.8
***Family History***		
Yes	167	16.7
No	826	82.4
Unknown	9	0.9
***Multi-primary Melanoma***		
Yes	152	15.2
No	849	84.7
Unknown	1	0.1
***Stage at Diagnosis***		
0	58	5.8
I	504	50.3
II	235	23.4
III	169	16.9
IV	9	0.9
Unstagable^¥^	27	2.7
***Current Stage***		
0	56	5.6
I	435	43.4
II	159	15.9
III	217	21.7
IV	129	12.9
Unstagable^¥^	6	0.5
***Primary Clark Level***		
I	58	5.8
II	108	10.8
III	150	14.9
IV	489	48.8
V	69	6.9
Unknown	128	12.8
***Thickness (mm)***		
*In situ*	58	5.8
< 1.01	321	32.0
1.01 – 2.00	272	27.1
2.01 – 4.00	161	16.1
> 4.00	121	12.1
Unknown	69	6.9
***Tumor Infiltrating Lymphocytes (TILs)***		
Absent	206	20.6
Non-brisk	397	39.6
Brisk	31	3.1
unknown	368	36.7
***Distant Metastasis***		
Yes	130	13.0
No	870	86.8
N/A^ϕ^	2	0.2
***Intransit Metastasis***		
No	942	94.0
Yes	27	2.7
N/A^ϕ^	33	3.3
***Number of Moles***		
None	260	25.9
Few	498	49.7
Moderate	169	16.9
Many	40	4.0
N/A^ϕ^	35	3.5
***Phenotypic Index***		
1 (low risk)	38	3.8
2	210	21.0
3	317	31.6
4	328	32.7
5 (high risk)	105	10.5
N/A^ϕ^	4	0.4
***Site of the Primary Melanoma***		
Extremities	535	53.4
Trunk	342	34.1
Head and Neck	71	7.1
Non-cutaneous^ω^	12	1.2
Unknown	42	4.2

* With the exception of 'current stage', the variables were recorded at the initial diagnosis.

^¥ ^patients with missing data on the 'T' classification.

^ω ^90% mucosal melanomas and 10% other sites.

^ϕ ^N/A: data not available.

### Biospecimens

Buccal cells were collected from mouthwash or buccal swabs (n = 985). Blood was also obtained from some individuals (n = 17). DNA from buccal cells was extracted using Puregene^® ^kits (Gentra Systems Inc., Minneapolis, USA), and blood was extracted with the QIAamp DNA Blood kit (QIAGEN Inc. Valencia, USA) using manufacturer's recommendations. DNA concentration was measured by spectrophotometry at 260 nm in a Spectramax Plus 384 (Molecular Devices, Sunnyvale, USA). The DNA quality was determined by the ratio A260/A280.

### Genotyping

The polymorphism MMP3 -1171 5A/6A were studied by fragment size analysis as previously described by Zinzindohoue [18]. The MMP2 substitutions were assessed by melting temperature analysis using the LightTyper instrument (Roche Applied Science, Indianapolis, USA) [19]. Briefly, 10–20 ng of genomic DNA were amplified using 0.5 units of AmpliTaq DNA polymerase (Applied Biosystems, Foster City, USA), 1.5 mM MgCl2, 1× PCR buffer, 200 μM dNTPs (Invitrogen, California, USA), 0.5 μM of each primer (forw: 5'-CTTTCTTCTCCAGTGCC-3'; rev: 5'-CCCTAAACTAGTAAAGAC AATCA-3' for MMP2 -1306 C/T; or forw: 5'-CAGTGGGGTCTTTGTGACCT, rev: 5'-GCGTTAGAGACGTTGGAACC-3' for MMP2 -735 C/T), and 0.2 μM of probe (5'-fluorescein-CCCAGCACTCCACCTCTTT-3' for MMP2 -1306 C/T; or 5'-fluorescein-GAATGCGGACCCTCCTGG-3' for MMP2 -735 C/T). Amplified samples were heated at 95°C for 2 min, cooled down to room temperature, and placed into the LightTyper instrument. Samples that failed were repeated once or twice as needed. All experiments included known controls and blanks. Genotyping of 5 to 10 % random selected samples was done as quality control and the results were read by 2 independent laboratory members.

### Sequencing

Direct sequencing was done in samples that showed unclear genotyping profiles. Briefly, samples were PCR amplified and then purified with a Qiagen purification kit following the manufacturer's recommendations (QIAGEN Inc., Valencia, USA). One to 10 ng of each purified sample were sequenced in the DNA Sequencing Core Facility at Memorial Sloan-Kettering Cancer Center. Samples were run in an ABI 3730-XLDNA Analyzer (Applied Biosystems, Foster City, USA). Sequencing electropherograms were read at least twice, reviewed manually and with the Mutation Surveyor software, version 2.41 (SoftGenetics LLC, State College, USA).

### Bioinformatics

The University of California Santa Cruz (UCSC) Genome Browser Database  was used to evaluate whether the undescribed mutations lay on conserved regulatory sequences and to determine empirically the functionality of the new variation.

### Statistical analysis

Two-sided Chi-Square tests, Cochran-Armitage tests for trend, and Fisher's exact tests were performed to test for association between genotype and various clinical and epidemiologic factors. Associations were examined in three different ways: comparing the homozygote high-transcriptional-activity allele group versus those having at least one copy of the low-transcriptional-activity allele, comparing the homozygote low-transcriptional-activity allele group versus all others, and looking at all three genotypes separately. Multivariate analyses were conducted using logistic regression, adjusting for age, sex, phenotypic index, moles, freckles, and race. Associations between number of high-transcriptional-activity alleles and the clinical and epidemiological variables were examined using the Chi-square and Kendall's Tau tests. Associations were considered significant when p < 0.050. To investigate associations between SNP and overall survival, time was measured from initial date of diagnosis with melanoma to date of death or last follow-up. Potential associations between genotypes and time to recurrence, defined as a patient's first recurrence of melanoma (local, intransit, nodal and/or systemic), and time to disease progression defined as progression to stage III or IV, were also examined. Survival estimates were computed using the methods of Kaplan and Meier and comparisons between genotypes were made using the log-rank test. All statistical analyses were carried out using SAS version 9.1 (SAS Institute, Cary, NC).

## Abbreviations

AJCC, American Joint Committee on Cancer ; CI, confidence interval; CMM, cutaneous malignant melanoma; dbSNP, SNP database from the NCBI; ECM, extracellular matrix; MMP, matrix metalloproteinase; OR, odds ratio; SNP, single nucleotide polymorphism; TILs, tumor infiltrating lymphocytes; UCSC, University of California Santa Cruz.

## Competing interests

The author(s) declare that they have no competing interests.

## Authors' contributions

JC carried out the genotyping, participated in the selection of SNPs, analysis, and prepared the manuscript; PR and SS participated in the genotyping; AP coordinated the patients' accrual and updated the clinicopathological and epidemiological database; NI performed the statistical analysis and contributed to the materials and methods section; DC, AH and AH contributed with subject accrual and discussions; KB contributed with pathology review; BR and CS participated in the analysis and interpretation of the results obtained with the *in-silico *methods; MB participated in the design and discussions; IO conceived and coordinated the study, participated in its design, analysis, discussion of results, and in the preparation of the manuscript. All authors read and approved the final manuscript.
